# Systemic Dental Pulp Stem Cell Secretome Therapy in a Mouse Model of Amyotrophic Lateral Sclerosis

**DOI:** 10.3390/brainsci9070165

**Published:** 2019-07-14

**Authors:** Junmei Wang, Kirstin Zuzzio, Chandler L. Walker

**Affiliations:** 1 Department of Biomedical Sciences and Comprehensive Care, Indiana University School of Dentistry, Indianapolis, IN 46202, USA; 2 Neuromuscular Research Group, Richard L. Roudebush Veterans Affairs Medical Center, Indianapolis, IN 46202, USA

**Keywords:** amyotrophic lateral sclerosis, ALS, dental pulp stem cells, DPSC, secretome, conditioned medium

## Abstract

Amyotrophic lateral sclerosis (ALS) is a devastating motor neuron (MN) disease with no cure. Accumulating evidence indicates ALS involves a complex interaction between central glia and the peripheral immune response and neuromuscular interface. Stem cell secretomes contain various beneficial trophic factors and cytokines, and we recently demonstrated that administration of the secretome of adipose-derived stem cells (ASCs) during early neuromuscular junction (NMJ) denervation in the mutant superoxide dismutase (mSOD1^G93A^) ALS mouse ameliorated NMJ disruption. In the present study, we hypothesized that administration of dental pulp stem cell secretome in the form of conditioned medium (DPSC-CM) at different stages of disease would promote NMJ innervation, prevent MN loss and extend lifespan. Our findings show that DPSC-CM significantly improved NMJ innervation at postnatal day (PD) 47 compared to vehicle treated mSOD1^G93A^ mice (*p* < 0.05). During late pre-symptomatic stages (PD70-P91), DPSC-CM significantly increased MN survival (*p* < 0.01) and NMJ preservation (*p* < 0.05), while reactive gliosis in the ventral horn remained unaffected. For DPSC-CM treated mSOD1^G93A^ mice beginning at symptom onset, post-onset days of survival as well as overall lifespan was significantly increased compared to vehicle treated mice (*p* < 0.05). This is the first study to show therapeutic benefits of systemic DPSC secretome in experimental ALS, and establishes a foundation for future research into the treatment effects and mechanistic analyses of DPSC and other stem cell secretome therapies in ALS.

## 1. Introduction

Amyotrophic lateral sclerosis (ALS) is a fatal neurodegenerative disease that causes death of upper and lower motor neurons in the central nervous system (CNS). The result is progressive skeletal muscle paralysis leading to respiratory failure, with an average survival time of 3–5 years post-diagnosis [1]. At the time of diagnosis, the disease has typically progressed to advanced stages, and the facts that few treatments and no cures for ALS have been developed are due in no small part to these aspects of disease presentation. Recently, additional focus has been placed on understanding the pathology that precedes symptom onset to obtain a more thorough understanding of potential biomarkers and targets for therapy. Much ALS research has focused on the lower motor neuron as a target of therapy, despite nearly two decades of research related to pre-symptomatic peripheral neuromuscular junction (NMJ) disruption. This “die-back” perspective as a potential initiator (or at least a key player) in disease progression, has gained traction with accumulation of evidence [2,3,4,5]; however, the process as a whole is dynamic and complicated. Within the CNS, it is now clear that astrocytes and microglia also affect motor neuron dysfunction and degeneration [6,7,8,9], which contributes to the current view of ALS as a highly complex disease with both temporal and cellular influences outside the MN as major contributors to disease onset and progression.

Recent research has shed light on possible therapies that prevent pre-symptomatic NMJ disruption as well as post-symptomatic motor neuron survival and extended lifespan in the classic mutant superoxide dismutase 1 (mSOD1^G93A^) of ALS [10]. It is clinically relevant to develop and test new therapies at symptom onset in pre-clinical studies, as currently, patients are treated once the disease is diagnosed. However, the lack of effective treatments speaks to the possible inability of therapies to improve patient function and survival at such advanced stages of disease. Therefore, studying progression of the disease to identify key pathologic events and targets for therapy is important for earlier disease detection and implementation of effective therapies. Still, it is clear that single agent therapies or individual cell targeting is not likely to advance therapeutic possibilities for the treatment of ALS.

Our recent research and that of our colleagues demonstrated the therapeutic potential of systemic administration of conditioned medium containing the trophic and other secretions from human adipose-derived stem/stromal cell cultures (ASC-CM). We showed that early pre-symptomatic treatment with ASC-CM reduced early NMJ denervation when administered before and during this early disease component [11]. Our colleagues previously demonstrated the post-symptomatic benefits of ASC-CM on spinal cord astrogliosis, MN survival and overall lifespan in the mSOD1^G93A^ mouse [12]. As such, the secretome from stem cell cultures is a promising and previously unexplored therapy in ALS. Dental pulp cells share many features with other stem cells, yet are derived from the ectoderm along with the central and peripheral nervous system [13,14]. Derived from neural crest cells, DPSCs are potentially uniquely valuable as therapeutics for disorders of the nervous system, as demonstrated in other disorders of the CNS and PNS [15,16,17]. Recently the secretome of DPSCs has also proven beneficial in models of Alzheimer’s disease [18] and spinal cord injury [19], and DPSCs are known to express valuable neurotrophic factors including neurotrophin-3 (NT-3), glial cell line-derived neurotrophic factor (GDNF), brain-derived neurotrophic factor, and nerve growth factor (NGF) [20,21]. The study presented here highlights pathology at three distinct time periods throughout disease onset and progression, and the therapeutic influence of systemic administration of dental pulp stem cell conditioned medium (DPSC-CM) on key aspects of disease progression in the mSOD1^G93A^ mouse model of ALS.

## 2. Materials and Methods

### 2.1. Culture of DPSCs and Preparation of DPSC Secretome

Human DPSCs (Cook General BioTechnology, Indianapolis, IN, USA) were harvested from third molars and characterized as previously described [22,23]. DPSC were cultured in low-glucose Dulbecco’s modified eagle medium (DMEM, Life Technologies, Inc., Carlsbad, CA, USA) containing 1 g/L D-glucose, L-glutamine and 110 mg/L sodium pyruvate, supplemented with 10% fetal bovine serum (FBS, Millipore, Billerica, MA, USA), 100 Units/mL penicillin, 100 µg/mL streptomycin and 0.292 mg/mL L-glutamine (Life Technologies, Inc., Carlsbad, CA, USA). The cells at passages 5–7 were grown to 80% confluency (approx. 2.8 × 10^6^/T75 flasks); then culture medium from each T75 flasks was changed to 10 mL low-glucose DMEM only and incubated for 72 hours. Following this incubation period, the conditioned medium was collected, centrifuged at 1000 g for 5 min to eliminate debris and filtered through 0.22 µm sterile filter (Millipore, MA, USA). The collected secretome was frozen at −80 °C in 1 mL aliquots for use in this study.

### 2.2. Animals and Animal Treatment

**Figure 1:**
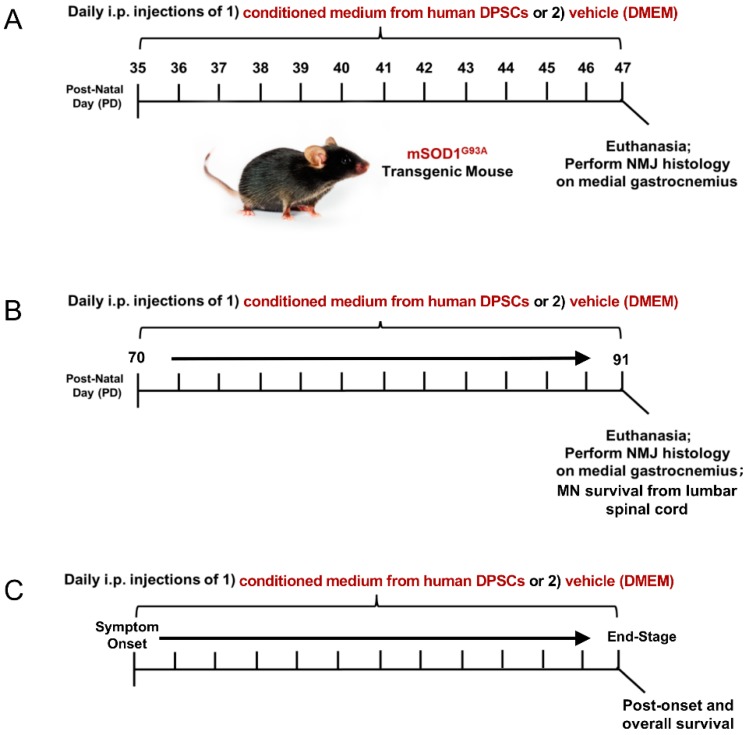
Experimental design and time-course of treatment and analysis. Wild-type, vehicle, and DPSC-CM treated mSOD1^G93A^ mice were designated for assessment at either (**A**) an early pre-symptomatic disease stage (PD35–47), (**B**) late pre-symptomatic (PD70–91) stage, or (**C**) from symptom onset (PD100–110) to end-stage (PD110–134). Upon sacrifice, histologic analysis of NMJ innervation of the gastrocnemius, MN survival in the lumbar spinal cord and survival outcomes were performed dependent on time period of treatment.

Transgenic mice (B6SJL-Tg (SOD1G93A)1 Gur/J, Jackson Laboratory) expressing the human superoxide dismutase 1 (mSOD1^G93A^) mutation were used in the study. All animal procedures in this study was approved by the Institutional Animal Care and Use Guideline of Indiana University. The mSOD1^G93A^ ALS mice were randomly assigned to a DPSC treatment group or a vehicle (DMEM) treatment group. The animals were then injected intraperitoneally (i.p.) with 200 µL DPSC secretome or vehicle (DMEM) at 3 different stages of disease: early pre-symptomatic, late pre-symptomatic, and at symptom onset to end-stage (Figure 1). End-stage was determined by the animal’s inability to right themselves after 30 seconds after being placed on its side. At this point, the mice were euthanized with an appropriate approved overdose of ketamine/xylazine followed by intracardial perfusion as described below.

To investigate whether DPSC secretome therapy could promote NMJ preservation in the early pre-symptomatic stage, 3 mSOD1^G93A^ ALS mice were treated by DPSC secretome at postnatal day 35–47 (PD35–47), as well as 3 ALS mice treated with vehicle (DMEM), compared with 3 wild type (WT) mice. To investigate whether DPSC could prevent motor neuron loss and NMJ denervation later in disease progression, DPSC-CM group (*n* = 6), vehicle group (*n* = 5) and WT group (*n* = 3) were treated at late pre-symptomatic stages (PD70–PD91). To detect whether DPSC therapy could extend lifespan, 9 mSOD1^G93A^ ALS mice were treated with DPSC-CM beginning at symptom onset through to end-stage, compared with 8 untreated mSOD1^G93A^ ALS mice.

### 2.3. Tissue Preparation

Depending on the period of treatment, animals were sacrificed at PD47, PD91 and end-stage (humane endpoint). Mice were anesthetized and perfused with 0.01M phosphate buffered saline (PBS, pH 7.4) followed by 4% paraformaldehyde (PFA) for tissue fixation. Spinal cords and hindlimb gastrocnemius muscles were dissected. Then, spinal cords were post fixed overnight at 4 °C and gastrocnemius muscle tissue was post fixed for 30 min at room temperature using the same fixative. Next, all the tissues were transferred to 30% sucrose solution for cryopreservation. Then, lumber spinal cord segments 1–3 (about 0.5 mm) and right medial gastrocnemius muscle were embedded in Optimal Cutting Temperature (OCT) compound (Tissue Plus, Fisher Healthcare, Houston, TX, USA) and serially cryosectioned at 20 µm by a cryostat (Leica, Wetzlar, Germany).

### 2.4. Gastrocnemius Muscle Weight

To weigh and compare the wet muscle weight of the gastrocnemius in WT, Vehicle, and DPSC-CM treated mice at PD91, mice were euthanized and fixed in 4% paraformaldehyde solution as described then rinsed in PBS. Excess moisture from the muscles was removed with paper towel, and both left and right gastrocnemius muscles were weighed on a scale. The weight recorded was the average of the two gastrocnemius muscle weights per mouse. Following weighing, the muscle tissue was further processed and prepared for cryosectioning and neuromuscular junction labeling.

### 2.5. Neuromuscular Junction Labeling and Quantification

Neuromuscular junction (NMJ) labeling and analysis of innervation was performed as previously described [11]. Briefly, 8 serial sections separated by an interval of 100 μm was incubated in 10% normal goat serum in PBS + 0.1% Triton-X100 (PBST) for 1 hour at room temperature followed by incubation with chicken anti-neurofilament primary antibody for labeling axonal innervation (1:500, Cat. #NFH, Aves Inc., Tigard, OR, USA) overnight at 4 °C. Slides were then washed in PBST and incubated with primary goat anti-chicken Alex 488-conjugated secondary antibody (Cat. #103-545-155, Jackson Immunoresearch Laboratories, West Grove, PA, USA) and Alex 555-conjugated α-bungarotoxin (1:800, Cat. #B35451, Life Technologies, Inc., Carlsbad, CA, USA), which labels the post-synaptic acetylcholine receptors (AchRs) of the NMJ, for 1 hour at room temperature. The slides were then washed in PBST and mounted for microscopic imaging.

To quantify innervated NMJs, five random high-power fields per section were selected from neurofilament/α-bungarotoxin co-labeled NMJs. Intact NMJs (yellow) were identified as those that exhibited an overlay of α-bungarotoxin (red) and neurofilament (green). Denervated NMJs were defined by labeling with only α-bungarotoxin (red). Percent intact NMJs was calculated as the number of intact NMJs (yellow)/total number of motor end plates (yellow + red). All slides were imaged and quantified by an individual blinded to the treatment conditions. Imaging was performed using a Nikon Eclipse Ti epifluorescent microscope and Nikon Elements software, and NMJ quantification was performed using NIH ImageJ software (National Institutes of Health, Bethesda, MD, USA).

### 2.6. Motor Neuron Immunolabeling and Quantification

For motor neuron immunostaining and quantification, spinal cord sections from mice sacrificed at PD91 were used and prepared via slightly modified methods from our previous publications [24,25]. In brief, 12–15 sections per mouse across 400 µm of the lumbar spinal cord were selected and incubated with primary rabbit monoclonal anti-NeuN antibody (1:500, Cat. #12943, Cell Signaling, Inc., Beverly, MA, USA) to label neurons. After incubation with secondary goat anti-rabbit Alexa Fluor 488 antibody (1:200, Cat. #111-545-045, Jackson Immunoresearch Laboratories, West Grove, PA, USA), neurons in the ventral horn were imaged using a Nikon Eclipse Ti epifluorescent microscope and Nikon NIS-Elements software (Nikon Instruments, Inc., Melville, NY, USA). Nuclei were labeled with Hoechst 33342 (bisbenzimide H 33342 trihydrochloride, Cat. #B2261, Sigma-Aldrich, St. Louis, MO, USA). For quantification of ventral horn MN, only large morphologically-intact neurons with a diameter ≥25 µm and distinct cell nucleus were counted per section. Small or injured neurons in the ventral horn with fragmented soma were not counted.

### 2.7. Immunofluorescence Labeling and Analysis of Spinal Cord Ventral Horn Glial Response

To assess central glia response after DPSC-CM treatment, immunofluorescence labeling of astrocytes and microglia was performed as previously described [24,26], on tissues collected at PD91 from late pre-symptomatic mSOD1^G93A^ mice. In brief, tissue was prepared and sectioned as described for NMJs, and 12–14 sections per mouse of every 400 µm spinal cord were selected to perform immunolabeling for glial reactivity. Sections were blocked by 10% goat serum in PBST for 1 h at room temperature, to block non-specific labeling. Next, the sections were incubated in the following primary antibodies: chicken anti-glial fibrillary acidic (GFAP, 1:500, Aves Inc., Cat. # GFAP) and rabbit anti-ionized calcium-binding adapter molecule 1 (Iba1, 1:1000, Wako, Japan, Cat. # 019-19741), overnight at 4 °C to label reactive astrocytes and microglia, respectively. The following day, the tissue sections were washed and incubated with appropriate secondary antibody: 488-conjugated goat anti-chicken or AlexaFluor 594-conjugated rabbit (1:200, Jackson Immunoresearch, Cat. # 111-585-144) for 1 h at room temperature. After washes in PBST, all labeled tissue slides were coverslip mounted using Fluoromount-GTM (Life Technologies, Inc., Carlsbad, CA, USA) with Hoechst 33342 (Sigma-Aldrich) to label cell nuclei.

To quantify GFAP and Iba1 labeling, the percent area of positive labeling in the ventral horn was measured using Image J software. In brief, the gray matter area of the ventral horn was outlined from the selected sections with the uppermost boundary set at the bottom of the central canal in cross section. Next, threshold values were adjusted to obtained area of positive labeling and the percent area was measured. Average percent ventral horn GFAP+ and Iba1+ area per section was calculated and statistically compared between groups.

### 2.8. Survival Analysis

Kaplan-Meier survival curves were prepared and analyzed for mSOD1^G93A^ mice-treated with DMEM and DPSC-CM from symptom onset to humane end-stage. A log-rank Mantel-Cox test was used to calculate χ^2^ and *p* values between treatment groups in GraphPad Prism 7.0 software (GraphPad, Inc., San Diego, CA, USA).

### 2.9. Statistical Analysis

Statistical analysis was performed by using GraphPad prism 7.0 software (GraphPad, Inc.). All data were evaluated with a Shapiro-Wilk test of normality, which indicated that all data exhibited a normal distribution. Then, data between two groups were analyzed via a two-tailed unpaired Student’s *t*-test and data between multiple groups were analyzed by ANOVA and Newman-Keuls post hoc test. Differences between two groups were considered significant statistically when *p* < 0.05.

## 3. Results

### 3.1. Early Pre-Symptomatic DPSC-CM Therapy Ameliorates NMJ Denervation

**Figure 2:**
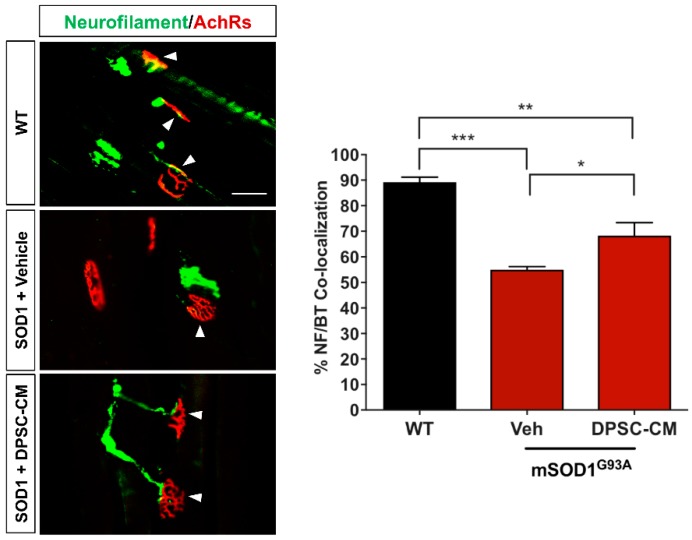
Early DPSC-CM therapy in mSOD1^G93A^ mice significantly reduced neuromuscular (NMJ) denervation. Administration of DPSC-CM from PD35–47 significantly increased innervated NMJs in the medial gastrocnemius (white arrowheads) compared to vehicle-treated mice. Data represent mean +/− SEM. Data analyzed via one-way ANOVA. *, *p* < 0.05; **, *p* < 0.01; ***, *p* < 0.001. (*n* = 3). Scale bar = 50 μm.

In our previous study and the research of others, innervated neuromuscular junctions in mSOD1^G93A^ mouse gastrocnemius significantly decrease between PD35 and PD47 [4,11]. In this study, we administrated DPSC-CM daily during this period to determine early NMJ protection (Figure 1A). Our data showed innervated NMJs with co-labeled neurofilament and α–bungarotoxin were significantly reduced in the vehicle-treated mSOD1^G93A^ mice compared with the WT group at PD47 (*p* < 0.001, Figure 2), while the DPSC-CM-treated group had significantly more intact innervated NMJs than the vehicle-treated group in the mSOD1^G93A^ mice (*p* < 0.05, Figure 2). This result indicated DPSC-CM administrating could prevent NMJ denervation in the early pre-symptomatic stage.

### 3.2. Late Pre-Symptomatic Treatment with DPSC-CM also Improved NMJ Innervation and Prevented Muscle Atrophy

**Figure 3:**
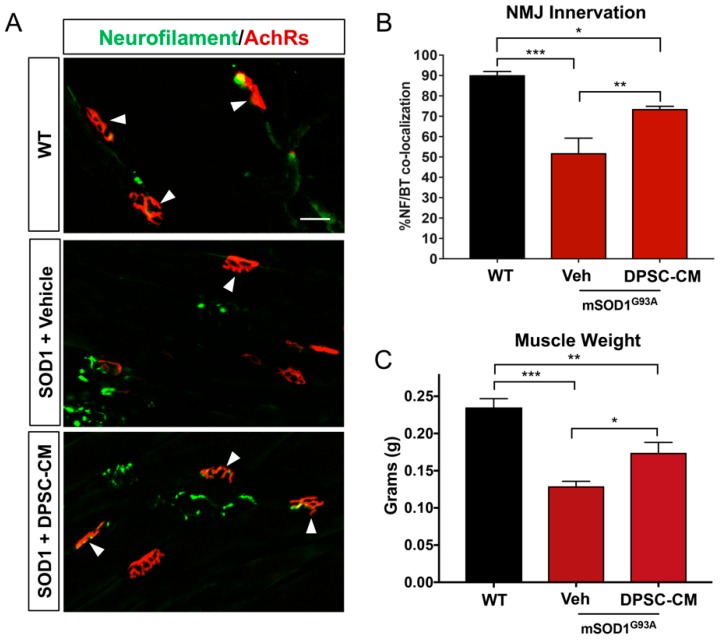
Late pre-symptomatic DPSC-CM therapy significantly improved NMJ innervation mSOD1^G93A^ mice. Administration of DPSC-CM from PD70–91 (**A**,**B**) significantly increased innervated NMJs in the medial gastrocnemius (white arrowheads), as well as (**C**) total wet muscle weight compared to vehicle-treated mice. Data represent mean +/− SEM. Data analyzed via one-way ANOVA. (WT, *n* = 3; mSOD1^G93A^, *n* = 5–6). *, *p* < 0.05; **, *p* < 0.01; ***, *p* < 0.001. Scale bar = 50 μm.

Improved NMJ denervation was also observed in the DPSC-CM treated mice compared to the vehicle treatment group at the late pre-symptomatic stage (PD91) (Figure 1B and Figure 3A,B) (51.9% ± 7.4%; *n* = 5) compared with WT mice (90.1% ± 1.8%; *n* = 3; *p* < 0.001, Figure 3B). Following DPSC-CM administration from PD70 to PD91, intact innervation NMJs were significantly increased in the DPSC-CM group (73.6% ± 1.3%; *n* = 6) compared with the vehicle group (*p* < 0.01, Figure 3B), indicating that DPSC-CM therapy also could improve NMJ innervation at the late pre-symptomatic stage. Wet muscle weights reflected the NMJ innervation quantification, with both the vehicle and DPSC-CM groups weighing less than WT gastrocnemius muscle (*p* < 0.001 & *p* < 0.01, respectively). As also shown with NMJ innervation at this stage, the gastrocnemius of DPSC-CM treated mice weighed significantly more than vehicle treated mice (0.17 ± 0.03 g vs. 0.13 ± 0.02 g, respectively; *n* = 5–6, *p* < 0.05, Figure 3C).

### 3.3. Late Pre-Symptomatic DPSC-CM Therapy Significantly Attenuated Ventral Horn MN Loss

**Figure 4:**
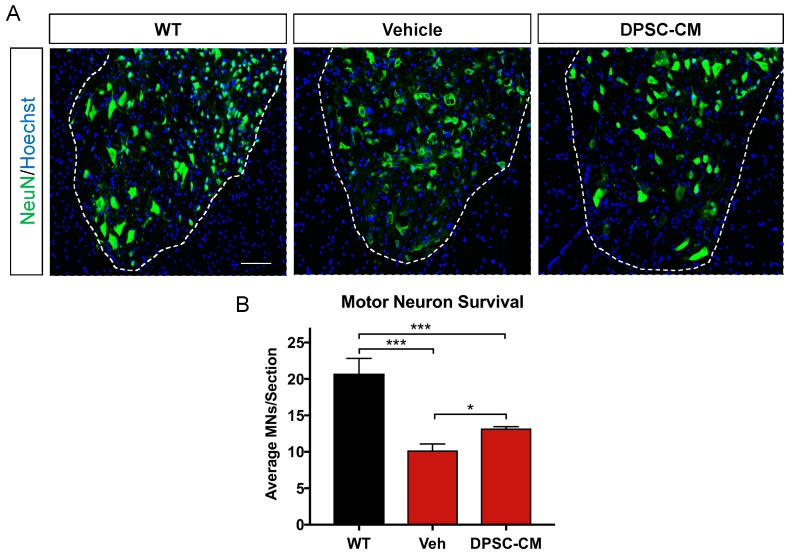
Late pre-symptomatic DPSC-CM treatment in mSOD1^G93A^ mice significantly attenuated ventral horn MN loss. Daily systemic DPSC-CM treatment from PD70–91 significantly increased MN survival in the mSOD1^G93A^ mouse lumbar spinal cord ventral horn compared to vehicle treatment, as measured by NeuN+ cells (green) in the ventral horn. Motor neurons in both Vehicle (Veh) and DPSC-CM treated mSOD1^G93A^ mice were significantly less than those in wild-type (WT) mouse lumbar spinal cord. Blue labeling indicates cell nuclei. Data represent mean +/− SEM. Data analyzed via one-way ANOVA. (WT, *n* = 3; mSOD1^G93A^, *n* = 5–6). *, *p* < 0.05; ***, *p* < 0.001. Scale bar = 150 μm.

Before symptom onset, MNs in the lumber spinal cord ventral horn already exhibit pathologic changes including vacuolation within the soma and proximal axons and progresses to dendrites and cell body by 78 days [27]. Our images showed MN in the mSOD1^G93A^ mice lumbar spinal cord ventral horn were injured or degenerating with a hole in the cell body (Figure 4A), so the number of normal MNs was decreased at late pre-symptomatic stage (PD91). Daily DPSC-CM treatment from PD70–91 (Figure 1C) significantly increased MN survival in the mSOD1^G93A^ mice lumbar spinal cord ventral horn, the number of healthy MNs increased significantly in the DPSC-CM treated group compared to vehicle treatment (*p* < 0.01, Figure 4B).

### 3.4. Late Pre-Symptomatic DPSC-CM Therapy Did Not Affect Glial Reactivity in the Ventral Horn of the mSOD1^G93A^ Mouse Spinal Cord

**Figure 5:**
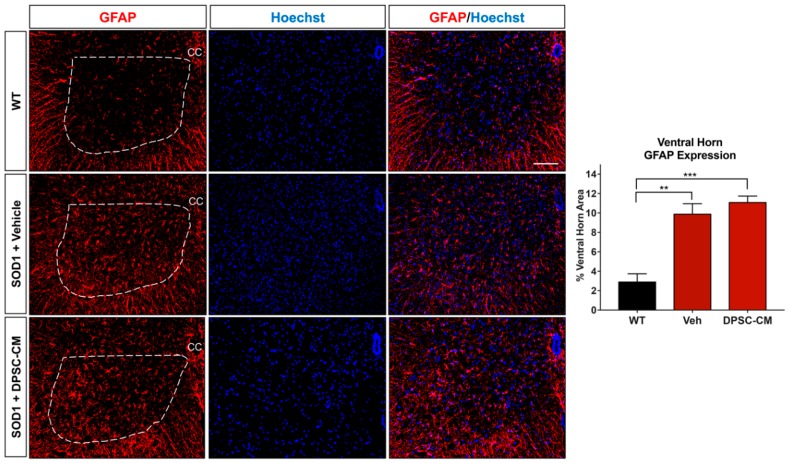
Astrocyte reactivity in the lumbar ventral horn of mSOD1^G93A^ mice was not significantly affected by late pre-symptomatic DPSC-CM therapy. Assessment of astrocyte reactivity via percent area of the ventral horn (dashed white line) occupied by GFAP+ labeling (red) showed no significant difference between DPSC-CM and vehicle treated mSOD1^G93A^ mice. Both treatment groups showed similar significant elevation of astrocyte reactivity during this stage of disease compared to WT mice. Data represent mean +/− SEM. Data analyzed via one-way ANOVA. (WT, *n* = 3; mSOD1^G93A^, *n* = 5–6). **, *p* < 0.01; ***, *p* < 0.001. CC = central canal. Scale bar = 100 μm.

**Figure 6:**
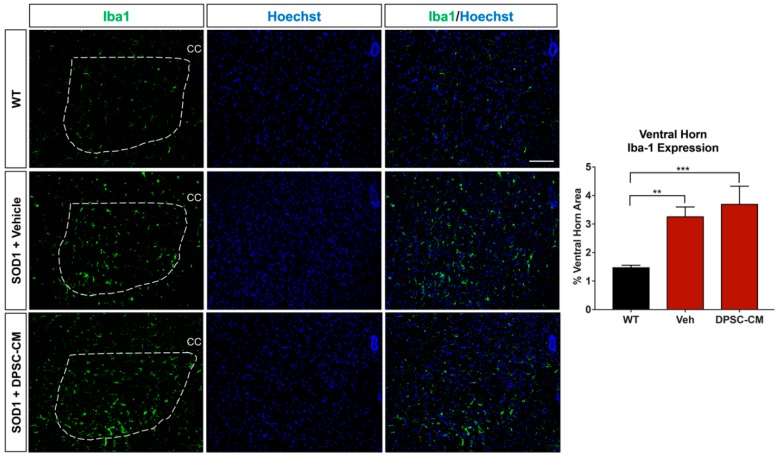
DPSC-CM therapy in the late pre-symptomatic period did not significantly alter lumbar ventral horn microglial reactivity. Microglial reactivity as measured by percent area of the ventral horn (dashed white line) occupied by Iba1+ labeling (green) was not significantly different between DPSC-CM and vehicle treated mSOD1^G93A^ mice at PD91. As with reactive astrogliosis, both treatment groups exhibited a similar significant elevation of microglial reactivity compared to WT mice. Data represent mean +/− SEM. Data analyzed via one-way ANOVA. (WT, *n* = 3; mSOD1^G93A^, *n* = 5–6). **, *p* < 0.01; ***, *p* < 0.001. CC = central canal. Scale bar = 100 μm.

To investigate whether DPSC secretome treatment affected astrogliosis and microglia reactivity, GFAP and Iba1 labeling were quantified in the ventral horn gray matter of the mSOD1^G93A^ mouse spinal cord at PD91 (Figure 5 and Figure 6). It has been demonstrated that glial reactivity is greatly increased in the central nervous system of mSOD1^G93A^ mice. Our results also showed that astrocyte and microglia reactivity increased dramatically in the ventral horn of the lumbar spinal cord in the vehicle group compared with the WT group (4.20 ± 2.14 and 1.48 ± 0.11 increased to 11.25 ± 0.18 and 3.27 ± 0.74, respectively (*p* < 0.01, *p* < 0.05; Figure 5 and Figure 6). No significant differences were observed in ventral horn astrogliosis and microglia reactivity through our comparison between DPSC-CM and vehicle treatment groups (*p* > 0.05, Figure 5 and Figure 6). These data indicated that DPSC-CM did not significantly increase or decrease glial reactivity in the ventral horn surrounding susceptible motor neurons.

### 3.5. Post-Symptomatic DPSC-CM Administration Significantly Extended Lifespan in mSOD1^G93A^ Mice

**Figure 7:**
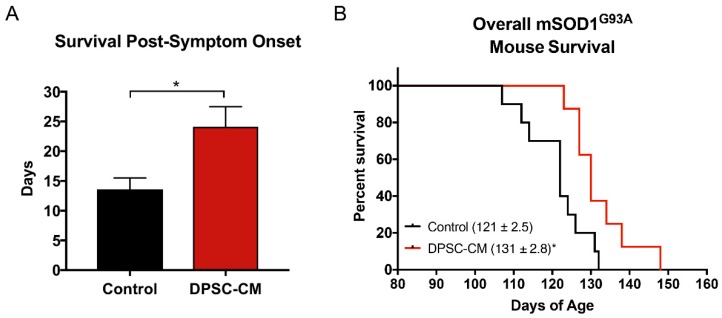
DPSC-CM treatment beginning at symptom onset significantly increased post-onset survival and overall lifespan of mSOD1^G93A^ mice. Daily DPSC-CM treatment from symptom onset until end-stage (point of humane euthanasia) significantly increased (**A**) post-onset survival and (**B**) extended overall lifespan in mSOD1^G93A^ mice by ~10 days compared to vehicle treatment. *, *p* < 0.05. (*n* = 8–9).

Transgenic mSOD1^G93A^ mice were treated with DPSC-CM from symptom onset until end-stage (point of humane euthanasia). The survival days from disease onset to death were measured. Our data was reported as days of post-onset survival (Figure 7A). Daily DPSC-CM treatment from symptom onset significantly increased post-onset survival (23.2 ± 2.5 days, *n* = 9) compared to vehicle treatment (13.2 ± 1.1 days; *n* = 8; *p* < 0.05). A Kaplan-Meier survival curve shows the overall lifespan of mSOD1^G93A^ mice in Figure 7B. DPSC-CM treatment also significantly extended overall lifespan (130 days; *n* = 9) in mSOD1^G93A^ mice compared to vehicle treatment (120 days; *n* = 8; *p* < 0.05; Figure 7B).

## 4. Discussion

The results of the present study demonstrate various benefits of systemic DPSC secretome administration at various stages of disease progression in the mSOD1^G93A^ mouse model of ALS. To our knowledge, this is the first study to show that (1) DPSC-CM can be therapeutic, and (2) that systemic delivery of stem cell secretome can ameliorate major aspects of disease progression through the lifespan in an ALS animal model. This study is our second report of the therapeutic value of human stem cell secretome in the mSOD1^G93A^ mouse model of ALS. The impetus for the study presented here was based on our initial findings that systemic conditioned medium from adipose-derived stem cells (ASC-CM) improved hindlimb neuromuscular innervation when administered during initial neuromuscular disconnection in the mSOD1^G93A^ mouse model [4,11]. To identify whether DPSC-CM could provide a similar therapeutic benefit, we repeated this study using DPSC-CM and found it also ameliorated NMJ denervation during this early period of pathology onset (PD35-47) (Figure 2).

The mechanism of how the secretome from DPSCs and ASCs may be mediating this benefit remains unclear. Likewise, whether the effect is due to central trophic influence on the motor neuron soma, in the periphery at the neuromuscular interface, or a combination of these and other effects is also unknown. Early in disease, it is possible that systemic stem cell secretome administration directly provides a therapeutic effect on spinal neurons or cells involved in the NMJ including Schwann cells and muscle cells. DPSCs are known to secrete various potent neurotrophic factors including NGF, BDNF, VEGF, and NT-3 that have known therapeutic effects in spinal cord injury (SCI) and other neurodegenerative diseases [20,21]; therefore, these factors could be directly protecting motor neurons, leading to their increased survival. However, such factors can also reach and stimulate peripheral glial cells and muscle cells, locally stimulating sustained innervation or reinnervation of NMJs [28,29,30]. Alternatively, the effects could be indirect through stimulating other cells or processes, including immunomodulation to influence cells of the spinal cord and muscle. Though more information is being brought to light by ongoing research concerning this early pre-symptomatic period of disease progression, there is evidence to support the possibility that conditioned medium may be acting on both [12,31,32].

Since NMJ disruption is one of the earliest pre-symptomatic anatomical characteristics in ALS, and the level of its progression can be reliably measured in the clinic through motor unit estimation (MUNE) and related assessments [33,34,35], we have a great interest in understanding not only the pathology and progression of this aspect of the disease, but also the potential therapeutic value in altering the course of its breakdown. Our prior study using ASC-CM was the first to show a therapeutic benefit of human stem cell secretome on early pre-symptomatic neuromuscular disconnection in an ALS model [11], and the present study demonstrated a similar benefit from DPSC-CM at this timepoint (Figure 2). Interestingly, despite considerably advanced progression of CNS pathology, gliosis, and MN loss at PD91, we observed a similar percentage of intact NMJ’s compared to that at PD47 (Figure 3). We anticipated that the loss of intact NMJs was a progressive process, where more NMJ disconnection occurred as the disease progressed. At this time point, we also observed significantly increased ventral horn MN with DPSC-CM treatment than vehicle-treated mice (Figure 4). An initial explanation might be that increased MN survival is responsible for decreased loss of innervated NMJs. However, evidence suggests this does not equate to reduced neuromuscular disconnection [3,36].

Recent research suggests that instead of a gradual progressive loss in NMJs once the process has started, recurrent bouts of compensatory sprouting as MNs that initially disconnect from NMJs also subsequently sprout branches from the retracted axons to form new NMJs [3]. Thus, NMJ loss is quite dynamic, and explains our finding that an approximately similar number of lost and preserved NMJs at PD91 was observed compared to early pre-symptomatic levels of loss in non-treated and treated mice, respectively. Ultimately, however, complete axonal degeneration occurs, and neurological deficits become evident in the 1–2 week period beyond the late pre-symptomatic period observed in our study.

To support innervation and a potential functional influence on the gastrocnemius, we found that DPSC-CM treated mSOD1^G93A^ mouse gastrocnemius muscle weighed significantly more than vehicle treated mice (Figure 3C), suggesting prevention of muscle atrophy. Whether this correlation is truly due to improved neuromuscular function will be explored in further studies. Still, the therapeutic effects on the NMJ at different stages of disease progression is intriguing, and cell specific influences, such as effects on the terminal Schwann cell or musculature, will be analyzed in future studies to better clarify the mechanism of this stem cell secretome-mediated therapeutic benefit.

Our colleagues demonstrated that administration of ASC-CM at symptom onset decreased spinal cord glial reactivity and inflammation, improved motor neuron survival and increased lifespan in the mSOD1^G93A^ mouse [12]. The evidence regarding astrocyte reactivity and MN survival following ASC-CM treatment strongly suggests that the beneficial components of the conditioned medium were acting centrally. This is reasonable, as the blood brain/spinal cord barrier is known to be permeable at least as early as 60 days of age in this mouse model [37]. In the present study, when we administered DPSC-CM daily on postnatal days 70–91 and at symptom onset, the secretome benefits on MN survival and improved lifespan could have resulted from direct central influence in the CNS microenvironment. If this is true, it is interesting that we found no significant difference in astrogliosis and microgliosis in the ventral horns of CM-treated and non-treated mice (Figure 5 and Figure 6). This finding suggests that the medium was affecting the motor neuron soma directly, and that it proved beneficial in this regard despite overall elevated gliosis in the gray matter surrounding the MN cell bodies. As the breakdown of the BSCB is pathologic, another therapeutic effect of systemic DPSC-CM during this period could be to repair the “leakiness” of the barrier. Mesenchymal stem cell secretions have been shown to improve outcomes by restoring BSCB integrity, as well as improved capillary density and enhanced axonal and myelin coherence in late stage mSOD1^G93A^ mouse spinal cord [38].

As astrocytes and microglia have been found to contribute to MN death and disease progression in ALS [39,40,41,42,43], perhaps the DPSC-CM provided a protective effect against glial influence via direct action on the MN. However, it is difficult to determine the exact influence of DPSC-CM on central glia, with complex phenotypes exhibiting (A1 and M1) inflammatory vs. inflammatory (A2 and M2) phenotypes of astrocytes and microglia, respectively, having an influence on neurodegeneration in ALS and conditions [42,44,45]. We did not look at designated markers of these phenotypes, so it cannot be excluded that DPSC-CM may have influenced the inflammatory status of these cells despite measures of overall reactivity remaining unaffected. Likewise, since we also observed improved NMJ innervation following DPSC-CM treatment during this time period, a combination of peripheral and central effects could have contributed to MN survival. Therefore, increased motor neuron survival may have been a reason for the increased number of intact NMJs. Further research is necessary to determine how and where in the body the stem cell secretome is imparting its benefits, and to better define the cell-specific influences of the treatment.

Our finding that the systemic administration of DPSC-CM from symptom onset until end-stage significantly increased survival (Figure 7), is the most clinically relevant of all the findings. This is because the vast majority of ALS patients with a non-hereditary form of the disease will only receive treatment once symptoms are noticeable and a diagnosis is made. Taken together with all of the pre-symptomatic therapeutic effects observed in mSOD^G93A^ mice in our study, particularly the prevention of MN loss in the late pre-symptomatic stage, the survival benefits strongly support the late-stage central effects of DPSC-CM. By end-stage, most lumbar MNs are lost, with cervical cord MNs on a rapid decline, but the NMJs have long been disconnected and MN axon degeneration has been established. Treatment between PD70–91 could still afford some peripheral influence, though central effects are likely. Given this therapy may impart some level of system-level pre-conditioning, recent studies suggest that homeostatic mechanisms may provide an underlying target for therapy and a possible reason why sporadic ALS patients might otherwise present with relatively fewer premorbid health conditions [46,47]. This is an interesting area of study that has not been extensively investigated but would be valuable and particularly pertinent to the patient population that our current study is intended to target.

## 5. Conclusions

In conclusion, this study provides a broad data set in relation to the central and peripheral influence of DPSC-CM as a treatment across different stages of disease progression in the mSOD1^G93A^ mouse model of ALS. Key areas of interest for future studies will focus on the components of the DPSC secretome, such as whether exosomes play a key therapeutic role, as well as to investigate the site of action at which DPSC secretome therapy has its effects across different disease stages. We believe this foundational study will set the stage for future comparison of the therapeutic benefits of different stem cell secretomes in the treatment of ALS.
